# Nonspecific complaints in the emergency department – a systematic review

**DOI:** 10.1186/s13049-020-0699-y

**Published:** 2020-01-28

**Authors:** Kirsi Kemp, Reija Mertanen, Mitja Lääperi, Leila Niemi-Murola, Lasse Lehtonen, Maaret Castren

**Affiliations:** 1 Department of Emergency Medicine and Services, Helsinki University Hospital, and Emergency Medicine, Helsinki University, Helsinki, Finland; 2 Department of Anesthesiology and Intensive Care Medicine, University of Helsinki and Helsinki University Hospital, Helsinki, Finland; 3 Department of Public Health, University of Helsinki and Helsinki University Hospital, Helsinki, Finland

**Keywords:** Emergency department, Mortality, Nonspecific complaint, Adult, Emergency services, Length of stay

## Abstract

**Background:**

Nonspecific complaint (NSC) is a common presenting complaint in the emergency setting, especially in the elderly population. Individual studies have shown that it is associated with significant morbidity and mortality. This prognostic systematic review draws a synthesis of reported outcomes for patients presenting with NSC and compares them with outcomes for patients presenting with a specific complaint.

**Methods:**

We conducted a literature search for publications, abstracts and conference presentations from Ovid, Scopus and Web of Science for the past 20 years. Studies were included which treated adult patients presenting to the Emergency Medical Services or Emergency Department with NSC. 2599 studies were screened for eligibility and quality was assessed using the SIGN assessment for bias tool. We excluded any low-quality studies, resulting in nine studies for quantitative analysis. We analysed the included studies for in-hospital mortality, triage category, emergency department length of stay, admission rate, hospital length of stay, intensive care admissions and re-visitation rate and compared outcomes to patients presenting with specific complaints (SC), where data were available. We grouped discharge diagnoses by ICD-10 category.

**Results:**

We found that patients presenting with NSC were mostly older adults. Mortality for patients with NSC was significantly increased compared to patients presenting with SC [OR 2.50 (95% CI 1.40–4.47)]. They were triaged as urgent less often than SC patients [OR 2.12 (95% CI 1.08–4.16)]. Emergency department length of stay was increased in two out of three studies. Hospital length of stay was increased by 1–3 days. Admission rates were high in most studies, 55 to 84%, and increased in comparison to patients with SC [OR 3.86 (95% CI 1.76–8.47)]. These patients seemed to require more resources than patients with SC. The number for intensive care admissions did not seem to be increased. Data were insufficient to make conclusions regarding re-visitation rates. Discharge diagnoses were spread throughout the ICD-10 main chapters, infections being the most prevalent.

**Conclusions:**

Patients with NSC have a high risk of mortality and their care in the Emergency Department requires more time and resources than for patients with SC. We suggest that NSC should be considered a major emergency presentation.

## Background

Nonspecific complaint (NSC) is a common presenting complaint in the emergency setting, especially for older adults [1, 2]. The concept of NSC is new, and its definition has not yet been formally established. Most commonly it has been defined by Nemec et al. “all complaints that are not part of the set of specific complaints or signs or where an initial working diagnosis cannot be definitively established” [3] or, as Djärv et al., “rapid decline of conscious patient’s own experience in mental and/or physical condition without signs or symptoms from a specific organ and without ongoing fever” [4]. In other words, NSC is defined as a lack of specific complaint. It has also been previously suggested that nonspecific complaint should be subsumed into the presentation of generalisedl weakness [5]. As with all emergency presentations, the duration of the symptom is crucial, separating newly developed nonspecific complaints from long-lasting geriatric symptoms such as frailty or incontinence [6].

Nonspecific presentations are common in the elderly due to physiological changes related to ageing [7–9]. The number of these presentations can be expected to grow with the ageing population. NSCs are easily overlooked in the highly stressful emergency setting, and more patients seem to fall in this category during times of high workload [10]. These patients have a median of four comorbidities [3] and the condition is associated with poverty [11], making these patients at risk of vulnerability. Individual studies have shown that their morbidity and mortality is high [3, 12, 13]. An acute serious condition is present in 51–59% of these patients [3, 14, 15]. The list of discharge diagnoses is long and heterogeneous, which makes differential diagnosis unusually wide for these patients [4].

Finding the correct diagnosis for NSC patients is time- and resource-consuming, and often cannot be completed in the emergency setting. Just 46% of NSC patients were discharged with the correct diagnosis from the ED [16]. The BANC study identified 12.2% of NSC as having drug-related problems, only 40% of which were correctly identified at the initial assessment [17]. All main categories of the ICD-10 were presented in the discharge diagnoses for NSC patients. In one study, diagnostic accuracy for NSC patients seemed to correlate with medical speciality, emergency medicine doctors and internists performing better than family physicians [18].

Different models have been tried for improving care for these patients, for example geriatric emergency departments or passing the ED straight to medicine for the elderly wards [7]. No single risk assessment instrument, frailty construct or risk factor has been shown to reliably predict outcomes for elderly patients in general [19]. While recognising the limitations of current evidence, a previous review article recommended a thorough history-taking and focused physical examination and advocated basic testing for patients presenting with generalised weakness [6]. The optimal pathway for care remains unclear. In order to create a structured approach for patients presenting with nonspecific complaints, knowledge on population characteristics and outcomes is required.

Many studies have compared patients presenting with organ- or illness-specific symptoms and nonspecific symptoms from the perspective of certain discharge diagnoses. In a Swedish study, median time to antibiotics for septic patients presenting with NSC was significantly longer than with SC [20]. Patients who develop septic shock often present as normotensive with vague symptoms [21]. In a recent study, 1 in 10 cancer patients received their diagnosis in the ED; common presentations included generalised weakness [22]. In another recent study, stroke was missed in 15.3% cases in general, but for NSC presentations, the rate went up to 64% [23]. Fire Department New York EMS care providers missed more than a third of stroke cases, atypical presentations significantly contributing to the field misdiagnoses [24]. These studies frequently show worse outcomes or diagnostic delay for nonspecific and atypical presentations. At the time of initial presentation, the final discharge diagnosis is yet to be established, which renders this type of study less useful for the emergency physician. We have therefore chosen to study the outcomes for patients presenting with virgin nonspecific complaints, from the perspective of initial contact.

This systematic review aims to draw a synthesis of NSC presenting in Emergency Medical Services (EMS) and the ED. We hypothesise that outcomes are worse for patients presenting with an NSC compared to patients presenting with an SC. Our primary objective is to show that patients presenting with NSC have increased mortality in comparison to patients presenting with SC, and thus, require urgent assessment and care in the ED. Our secondary objective is to compare ED length of stay, resource utilisation and re-visitation rates, as well as hospital admission rates, intensive care unit admission and hospital length of stay between patients presenting with NSC and SC. We hypothesise that outcomes for patients presenting with NSC are worse.

## Methods

We submitted the study protocol to Prospero prior to screening, and it was published during the screening process with the ID CRD42019123552 [25]. This study only utilises previously published and de-identified data from peer-reviewed studies, and thus it was not necessary for us to apply for ethical review board approval.

The pico for our study is as follows:

**P**opulation: Adult patients presenting to the Emergency Department with an NSC.

(**I**ntervention: Not applicable to a prognostic review)

**C**omparison: Adult patients presenting to the Emergency Department with an SC.

**O**utcomes: Mortality, Triage classification, ED length of stay, Admission, Hospital LOS, ICU admission rate, number of consultations, number of required resources, re-visitation rates.

### Data collection

We searched for publications, abstract and conference presentations on the topic, from the last 20 years, in English. The period of 20 years was selected due to rapid development in the field of emergency medicine in the past decades. Inclusion criteria included age ≥ 18 years and presenting with a nonspecific complaint to the EMS and ED.

We conducted our first literature search on 29 January 2019 in the following databases: Ovid, Scopus and Web of Science, including Web of Science conference proceedings. A librarian from Terkko Health Hub collaborated in the literature search [26]. Search details for the first database searches are presented in Additional file 1. Search terms are summarised in Table 1.

**Table 1:** Search terms for nonspecific complaints in the emergency setting

Any of the following	AND	Any of the following
Nonspecific complaint		Emergency Department
Weakness		
Decreased General Condition		
General Disability		
Off the legs		Emergency Medical Services
Not coping		
Lethargy		
Failure to thrive		
Home Care Impossible		
Acopia		
Anorexia		
Decreased mobility		

The first search resulted in 2020 records that we saved to the Mendeley reference manager. Two independent researchers screened the records for inclusion/exclusion criteria. After screening, we recognised further search terms, with which we conducted another two searches in the aforementioned databases on 13 February 2019, resulting in a further 76 records and 7 July, resulting in a further 542 records. After removing duplicates, 2226 records remained. These included three studies for which we were unable to find abstracts or full texts online. We requested these studies directly from their authors.

The first screening resulted in 100 abstracts that were reviewed in full text; of these, 88 failed to meet the inclusion criteria. Two independent researches assessed the bias of the 12 eligible studies using the Scottish Intercollegiate Network for cohort studies criteria [27] (Additional file 1). Any studies with low methodological quality were excluded, resulting in six eligible studies. Two studies were disagreed upon by the two initial assessors, resulting in exclusion by the third assessor.

**Fig. 1:**
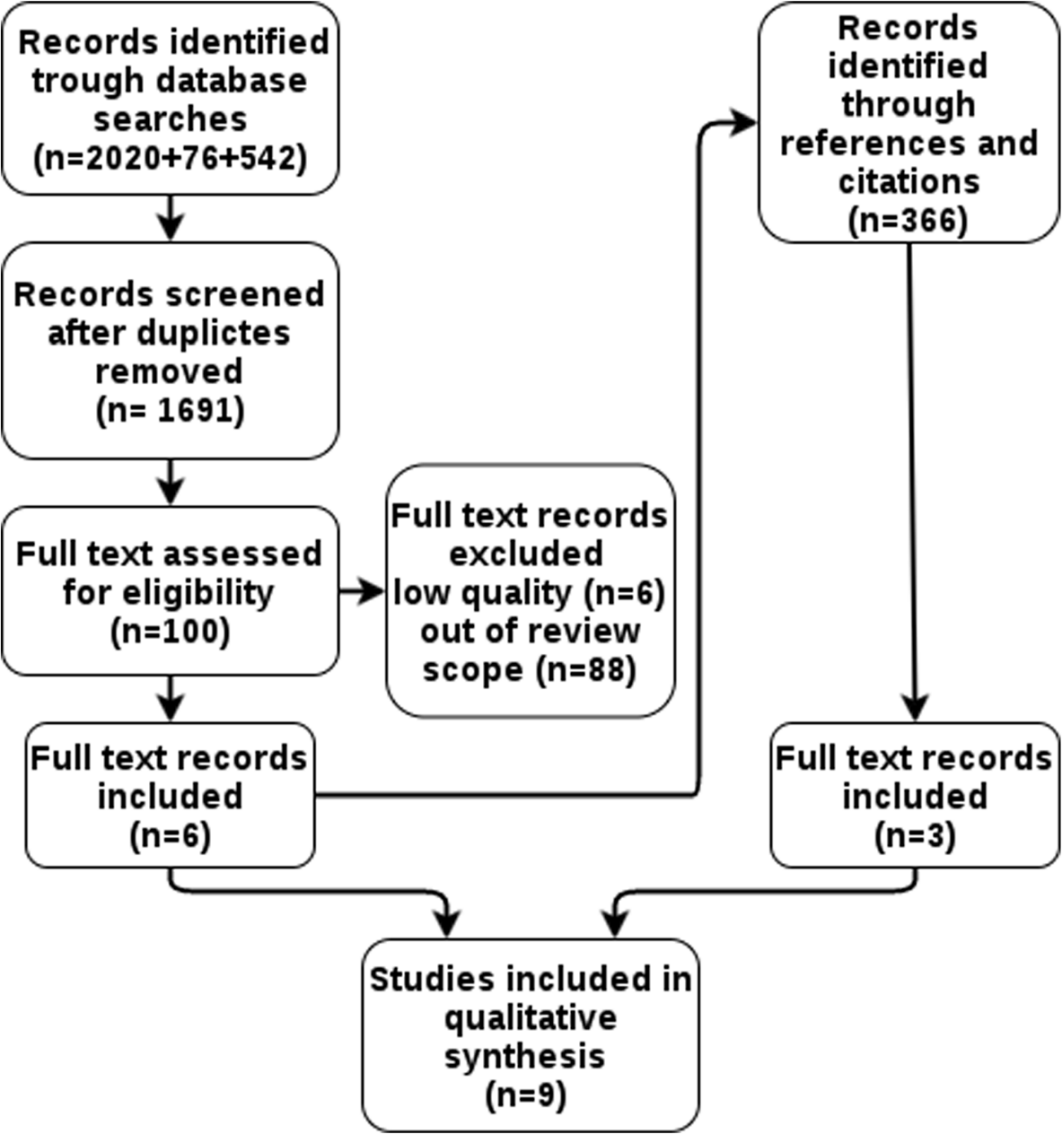
Prisma flow diagram for database searches

The references and citations of included articles were screened for eligibility, resulting in 366 further articles, three of which were included. In total, after removing duplicates, 2057 records were screened. Nine eligible studies of acceptable quality were included (Fig. 1). A list of potentially relevant studies that were excluded from this review is shown in Additional file 1.

Data from the included studies was extracted and saved to a summary table in Excel. Extracted data included:

1. Study characteristics: study setting, study location (emergency department or prehospital), median age, inclusion criteria, gender distribution, name of presenting complaint, number of participants in NSC and SC groups.
2. Outcomes with absolute figures and statistical parameters: Mortality with time of follow- up (i.e. in-hospital, 30-day), triage category, emergency department length of stay, admission rates, hospital length of stay, ICU admission rate, number of consultations, number of required resources (diagnostic tests and procedures), re-visitation rates with follow-up time.

### Analysis

We present the characteristics of the included studies by type, sample size, participant characteristics, presenting complaint and prevalence of nonspecific complaints. We conducted a meta-analysis of our primary outcome, in-hospital mortality, and reported other mortality figures individually.

Concerning triage category, we analysed the likelihood of being classified to an urgent triage category (1 or 2) between patients presenting with NSC and SC. We compared admission rate and in each included study and presented the result in a forest plot.

We compared emergency department length of stay and hospital length of stay in each included study and presented the results in text. The original studies had presented their data with statistical methods that were incomparable by statistical methods.

We found the number of other screened outcomes, required resources, rate of revisit and ICU admission rate insufficient for further analysis, and these results have been presented in a table and plain text.

Meta-analyses were done using a random-effects model to calculate pooled ORs and their 95% confidence intervals. A standard inverse-variance approach was used to estimate the log ORs. We felt that the studies were not identical enough to warrant the use of fixed effects. Furthermore, we wanted to make sure that the results were valid for generalization to other populations as well. We therefore chose the random-effects model instead of the fixed-effects model for pooling the data. As we did not have access to the original data and the included articles unfortunately did not have adjusted regression models available, we performed the meta-analysis using the unadjusted regression models. Analysis was conducted using R version 3.5.2 and the meta-analysis was done using the metaphor package [28, 29].⁠

We grouped the discharge diagnoses by ICD-10 category and gave frequencies for any specific diagnoses that were reported within those categories.

We assessed the quality of our method by filling out the PRISMA checklist (Additional file 1) [30]. The certainty of results was assessed with the GRADE method, by a team of three researchers [31].

## Results

### Characteristics

All studies show patients with nonspecific complaints to be older adults (Table 2). The prevalence of NSC in the adult population ranged from 1 to 2%. In older adults (≥65-year-old), prevalence ranged from 6.4 to 14%.

**Table 2:** Characteristics of included studies

Article	Study type	Presenting complaint	Definition for NSC	Population	NSC group	Control group	Total	Prevalence of NSC
Bhalla et al. 2019 [1]	Cross-sectional cohort	Weakness and fatigue	Listed in NHAMCS as generalised weakness 1020.0 or tiredness and exhaustion 1015.0	Age ≥ 65; All ED visits;	6.4% (~ 5.4 million); Age 65–74 years; 4.6%; 75–84 years: 6.5%; age ≥ 85: 7.7%	93.6% (~ 79 million)	85 million	1% of all adults; 6.40% of elderly
Djärv et al. 2015 [4]	Retrospective cohort	Decreased general condition	Adapt criteria for DGC	Age ≥ 18; All ED visits	1182; 54% female; 83% ≥65 years old; 57%; ≥80 years old	20,775; 52% female; 53% ≥65 years old; 27% ≥80 years old	21,957	1714 / 89,554; 1.9% of all adults
Lamantia et al. 2010 [32]	Retrospective cohort	Generalised weakness	Chief complaint independently by two reviewers according to reason for visit classification schema developed for use by the NHAMCS by national centre for health statistics	Age ≥ 75; All ED visits	n/a^b^	n/a	3188; 60.8% female; median age 82	n/a
Quinn et al. 2015 [33]	Retrospective cohort	NSC	As Nemec et al	Age ≥ 70; All ED visits	478; 54.8% female; Age avg. 81.9 years, SD 6.19	n/a	n/a	n/a
Safwenberg et al. 2007 [34]	Prospective cohort	General disability	As Djärv et al	Age ≥ 18; Non-surgical ED patients	719; median age 82	n/a	12,995; 51.5% female; median age 66	5.5% of adult non-surgical
Safwenberg et al. 2008 [35]	Prospective cohort	General disability	As Djärv et al	Age ≥ 18; Non-surgical ED patients	719; median age 82	n/a	12,995; 51.5% female; median age 66	5.5% of adult non-surgical
Sauter et al. 2018 [36]	Prospective observational^a^	NSC	As Nemec et al	Age ≥ 18; Admitted to medical ward from ED	165; 47.9% female; median age 71	546; 44.5% female; median age 69	711	n/a
Wachelder et al. 2017 [2]	Prospective observational	NSC	Not specified	Age ≥ 65; Internal medical patients at the ED	244; 50.4% female; Age mean 77.6 (SD 7.3)	1540; 54.4% female; Mean age 77.5 (SD 7.7)		244/2381 (9%) of adult medical patients; 244/1883 (14%) of elderly medical patients
Vilpert 2018 [37]	Retrospective cohort	Home care impossible	Lausanne Triage and priority scale category “home care impossible”	Age ≥ 65; ED visits excluding nursing home residents	5.5% home care impossible; 4.1% failure to thrive^a^	n/a	39,178; 54.6% female; Mean age 78.5 ± 7.9 years	9.6% of elderly community-dwelling

^a^Post-hoc classification can be questioned to be truly prospective, as the data were obtained previously, and the raters were not blinded to outcomes or diagnoses

^b^n/a: data not presented in original studies

Eligible studies were assessed for bias and we found the quality in all studies to be in the unacceptable or acceptable categories (Additional file 1). There were no studies that met the criteria for high quality. All nine included studies were observational. Five of the nine studies were retrospective and four were prospective. All the included studies took place in an emergency department setting. There was some heterogeneity within studies concerning study populations: five studies only included elderly patients, four included patients over the age of 18.

Three studies included only non-surgical internal medical patients and six studies included all emergency department patients.

All studies had a very low (down to 0) percentage of patients lost to follow-up. Due to the observational nature of the studies, blinding was not possible.

### Mortality

**Fig. 2:**
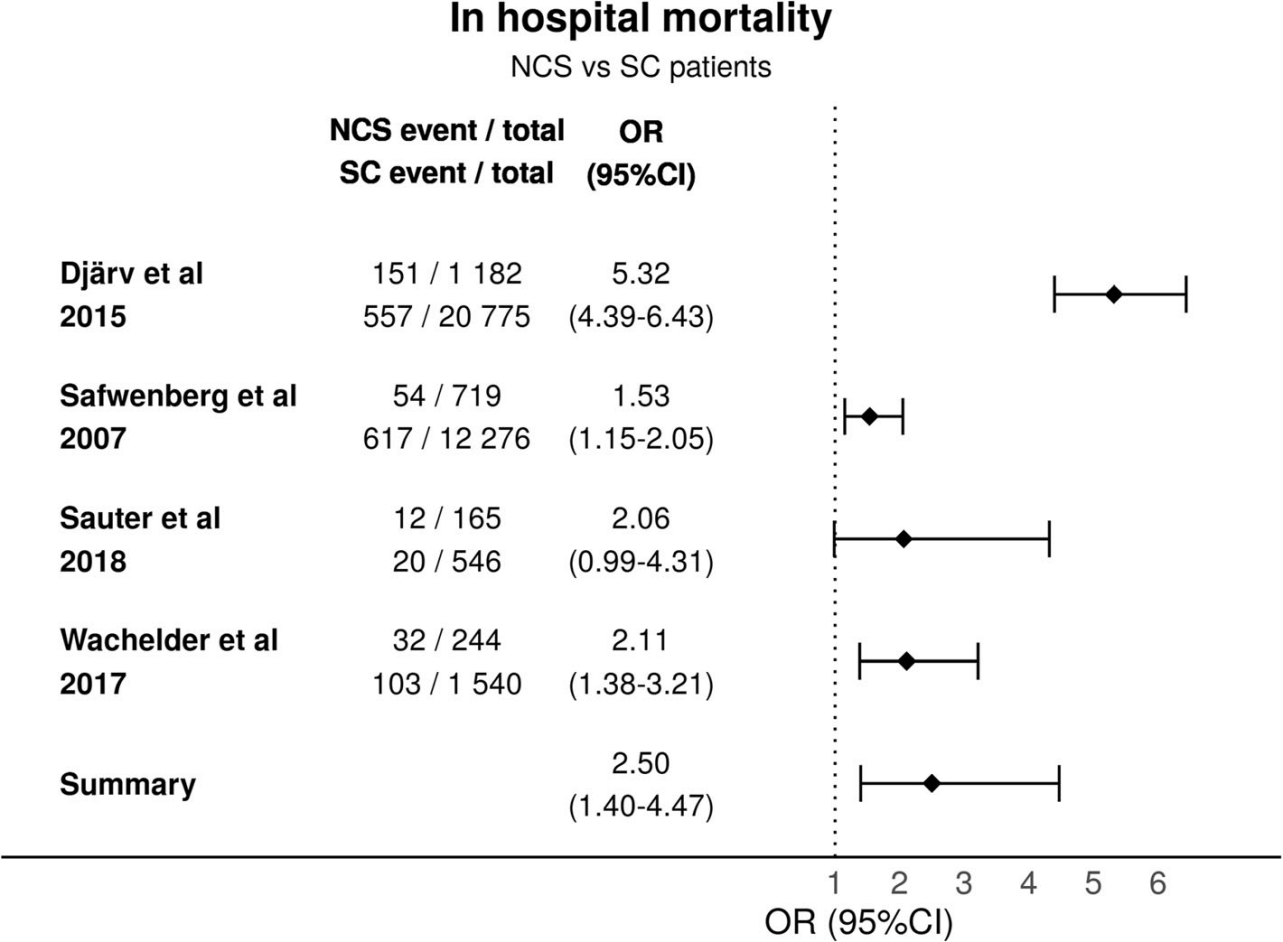
In-hospital Mortality

Five articles reported in-hospital mortality for patients with NSC. In-hospital mortality ranged from 7.3 to 15.6% in most studies. One study with very few admissions reported in-hospital mortality of 36.4%, there was no comparison group [33]. The studies were significantly heterogeneous (*p* < 0.001) and highly inconsistent (I^2^ = 91%). The variance of the true effect size was estimated to be 0.30 (T^2^). The heterogeneity between the studies is most likely due to different population differences. The summary odds ratio when comparing NSC to SC was 2.50 (95%CI (1.40–4.47)) (Fig. 2).

30-day mortality was assessed in one study: 20.1% of patients with NSC died within 30 days, compared to an 11.0% 30-day mortality for patients with SC [HR 1.7 (95% CI 1.2–2.4)] [34]. One study reported an increased 10-year mortality for NSC patients in comparison to SC [35]. According to GRADE [31] the certainty of evidence for mortality is high (Additional file 1).

### Triage category

**Fig. 3:**
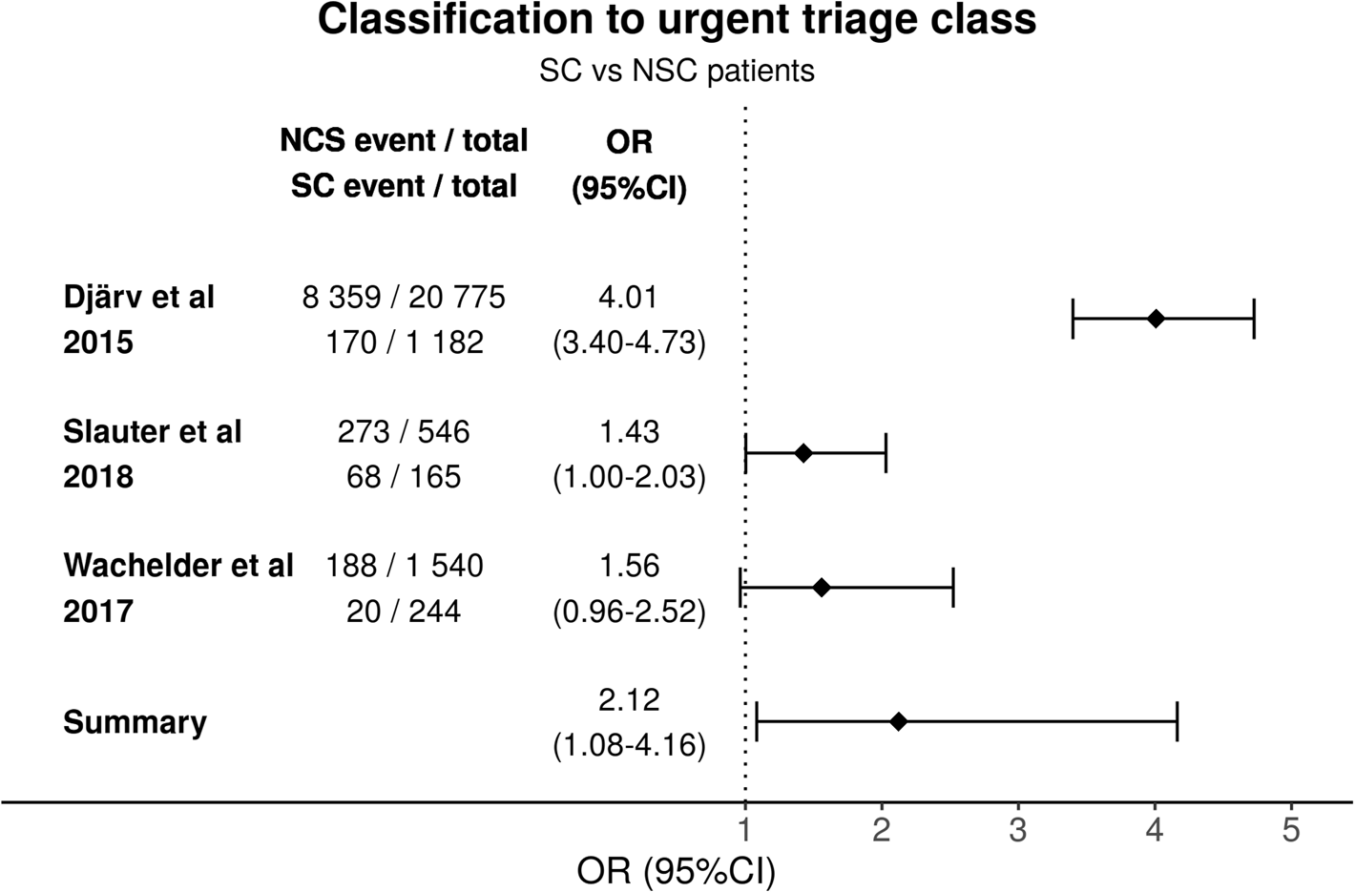
Comparison of classification to urgent triage class for patients presenting with SC and NSC

Three studies compared triage between NSC and SC patients. As with the mortality analyses the studies were once again significantly heterogeneous (*p* < 0.001) and the study differences contributed considerable amount to the total variation (I^2^ = 92%). The estimated variance of the true effect sizes was 0.32 (T^2^). The urgent triage class had a summary OR of 2.12 95%CI (1.08–4.16) when comparing the SC patients to NSC patients (Fig. 3). According to GRADE the certainty of evidence for triage is moderate (Additional file 1).

### Emergency department length of stay

Three studies reported emergency department length of stay. Two studies showed that it was significantly increased in patients with NSC in comparison to patients with SC: Bhalla et al. reported that ED length of stay increased from 249.4 (95% CI 240.3–258.4) to 299.6 min (95% CI 279.4–319.7), *p* < 0.0001 and Wachelder et al. reported a median increase from 178 (IQR 6–970) to 188 (IQR 23–421) minutes, *p* = 0.004 when patients presented with an NSC [1, 2]. Sauter et al. reported no increase between the groups: median stay for NSC patients was 6.27 h (IQR 3.11) and for SC patients was 6.09 h (IQR 3.26), p = 0,497 [36]. According to GRADE the certainty of evidence for ED length of stay is low (Additional file 1).

### Admission rate

**Fig. 4:**
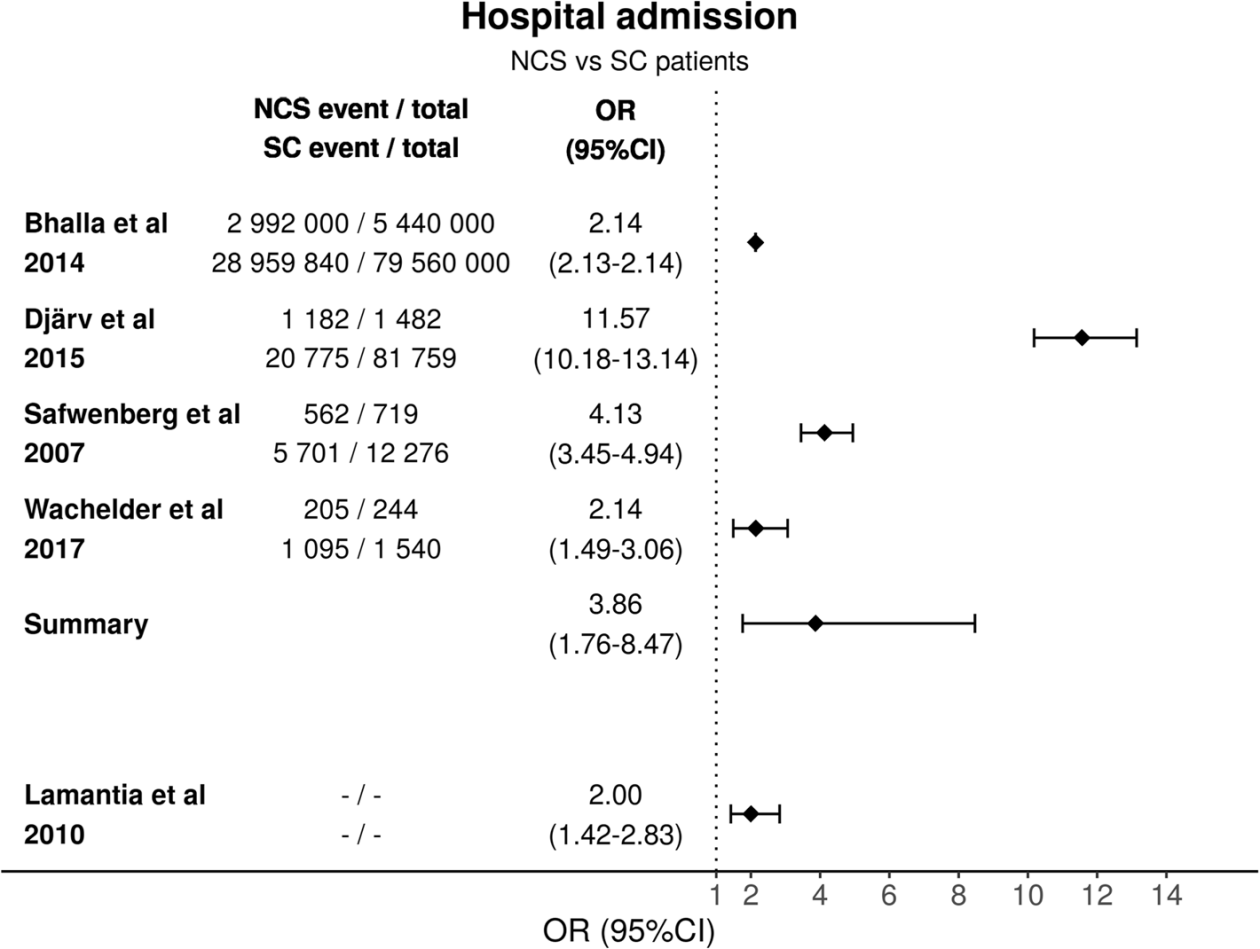
Comparison of hospital admission rate for patients presenting with NSC and SC

Admission rates for NSC were high (55–84%) in all but one study; Quinn et al. reported an admission rate of 2.3% [33]. There was significant heterogeneity between the studies (p < 0.0001) and the studies were highly inconsistent (I^2^ = 99%) in this comparison too. The estimated variance for true effect size was 0.64 (T^2^). The four included studies all had statistically significant differences between NSC and SC patients and the summary OR was 3.86 (95%CI 1.76–8.47) (Fig. 4). Vilpert et al. reported admission rates by age group: 79.6% for aged 65 to 84 SE (1.7) (95% CI 76.3–82.8) and 77.8% for aged over 85 SE (3.2) (95% CI 71.4–84.1) [37]. LaMantia et al. reported increased admission rates for NSC patients, OR 2.00 (95% CI 1.42–2.83), but they did not report their exact figures for events [32]. According to GRADE the certainty of evidence for hospital admission is moderate (Additional file 1).

### Hospital length of stay

Three studies compared hospital length of stay between NSC and SC patients. LOS was increased from one to three days in all studies. One study reported a hospital LOS of six days for NSC patients and four days for SC patients without statistical analysis, no confidence intervals were provided [34]. Two studies reported a significant increase in-hospital stay. Sauter et al. reported a median 6.51 days (IQR 5.85) versus 5.22 (IQR 5.85), *p* = 0,005 [36]. Wachelder et al. reported that patients presenting with and NSC had a median stay of 9 days (IQR 4–15) whereas patients presenting with a SC had a median stay of 6 days (IQR 2–12) [2]. One study reported a median NSC stay of five days, but no comparison to the SC group [33]. According to GRADE the certainty of evidence for hospital length of stay is low (Additional file 1).

### Other outcomes

Required resources, re-visitation rates and ICU admissions are summarised in Table 3. Bhalla et al. found a significant increase in the number of diagnostic tests, whereas Wachelder et al. did not report an increase. Bhalla et al. reported an increase in required procedures and Wachelder et al. reported an increase of need for consultations [1, 2].

**Table 3:** Other outcomes. Number of diagnostic tests and required procedures, need for consultations, return rates, ICU admission rates

Outcome		Study	NSC	SC	
Number of diagnostic tests		Bhalla et al.	7.7 (95% CI 7.3–8.1)	6.0 (95% CI5.7–6.2)	p < 0.0001
		Wachelder et al.	3.3 (1.7)	3.1 (1.8)	*p* = 0.1
Required procedures		Bhalla et al.	73.3% (95% CI69.5–76.8)	63.9% (95% CI 62.7–66.1)	p < 0.0001
Need for > 1 consultations		Wachelder et al.	19 (7.8%)	217 (14.1%)	*p* = 0.03
ICU admissions		Bhalla et al.	3.8% (95% CI 2.4–6.1)	3.51% (95% CI 2.9–4.3)	
		Wachelder et al.	2.5%	2.9%	*p* = 0.67
Return to ED	Within 30 days	LaMantia et al.	OR1.57 (95% CI 1.06–2.3)		*p* < 0.03
	Within 90 days	Wachelder et al.	57 (23.4%)	435 (28.5%)	HR 0.8 (95% CI 0.6–1.1)

Two studies reported an increase in re-attendance for patients with NSC. Re-visitation rates were increased at 30 and 90 days for NSC patients (Table 3) [1, 2]. One study reported a re-visitation rate of 16.9% for patients with NSC, but no comparison to the SC group [33].

Two studies compared ICU admission rates between patients with NSC and SC. Neither reported a significant increase (Table 3) [1, 2].

### Discharge diagnosis

Only four of the nine included studies reported the discharge diagnosis for NSC patients. Discharge diagnoses were distributed within all categories (Table 4), infections being the most prevalent.

**Table 4:** Discharge diagnoses by ICD-10 chapters

	Djärv (2)	Quinn (41)	Safwenberg (3)	Bhalla (1)
A00-B99	Certain infectious and parasitic diseases 8% • Sepsis 3% • Intestinal infectious disease 1%	–	Infection other than respiratory tract infection or urinary tract infections 7%	–
C00-D49	Neoplasm 9%	–	Cancer 10%	–
D50-D89	Diseases of the blood etc. 2% • Anaemia 1%	Anaemia 1.5%	–	Anaemia, unspecified 5%
E00-E89	Endocrine, nutritional, and metabolic disease 9% • Other disorders of fluid, electrolyte, and acid– base balance 3% • Volume depletion 1% • Diabetes mellitus 2%	Dehydration 5.6%	Fluid or electrolyte disturbance 7% Diabetes/endocrinology diagnosis 10%	Volume depletion 7% Dehydration 5%
F01-F99	Mental and behavioural disorder 5%	–	–	–
G00-G99	Diseases of the nervous system 5%	–	–	–
I00-I99	Diseases of the circulatory system 14% • Cerebrovascular diseases 6% • IHD 2% • AF 1% • Heart failure 2%	Stroke 4% TIA 10%	Stroke 8% Congestive heart failure 9%	Congestive heart failure, unspecified 7%
J00-J99	Diseases of the respiratory system 14% • Influenza and pneumonia 10% • Chronic lower respiratory disease 2%	Pneumonia 1.9%	Respiratory tract infection 16%	Pneumonia, unspecified 14%
K00-K95	Diseases of the digestive system 10% • Gastric ulcer 1% • Duodenal ulcer 2% • Other diseases of intestines 2% • Alcoholic liver disease 2%	Abdominal pain 4% Constipation 2.3%	Intestinal diseases 5%	Haemorrhage of the gastrointestinal tract 4%
L00-L99	Diseases of the skin and subcutaneous tissue 1%	–	–	–
M00-M99	Diseases of the musculoskeletal system and connective tissue 2%	–	–	–
N00-N99	Diseases of the genitourinary system 10% • Renal failure 2% • Cystitis 1% • Urinary tract infection NAS 6%	UTI 11.3%	Urinary tract Infection 14%	Urinary tract infection, unspecified 13%
R00-R99	Symptoms, signs, and abnormal clinical and laboratory findings, not classified elsewhere 7% • Dizziness and giddiness 1% • Malaise and fatigue 1% • Syncope and collapse 1%	Vertigo 2.3% Syncope 1.7%	–	Other malaise and fatigue 29% Syncope and collapse 11% Fever and other physiological disturbance 5%
S00-T88	Injury, poisoning, and certain other consequences of external causes 3%	Fall 4.1%	–	–
Z00-Z99	Medical observation and evaluation for suspected diseases and conditions 1%	Social issue 2.7%	Miscellaneous 9.1%	–

## Discussion

Our analysis demonstrates with high certainty that in-hospital mortality for patients presenting to the EMS with a nonspecific complaint is increased. The results indicate with moderate certainty that admission rates are high and that NSC patients are undertriaged. There seems to be low certainty that both the ED and hospital LOS are increased for patients with NSC, which has previously been found to increase both mortality and morbidity [38]. More evidence is required to assess resource utilisation, re-visitation rates and ICU admissions for patients with NSC.

Our mortality figures are in keeping with other studies in the field. Increased in-hospital mortality for septic patients presenting with NSC have previously been reported [39]. Increased mortality rates have previously been reported for NSC patients in the acute medical unit setting [40].

Admission rates were high in all studies except one. This might also apply to the prehospital environment: weakness has been shown as an independent predictor for hospital admission [41]. Similar results have been reported in a study that also included paediatric patients [42]. High admission rates, 82–84% for NSC patients triaged to ESI categories 2–3, have been reported in two studies [3, 15].

Our review shows that NSC patients are undertriaged, which is in keeping with previous findings for NSC patients with sepsis [20]. Rutschmann et al. have reported an under-triage rate of 26% [15]. Patients who are triaged as less urgent will have longer waiting times and longer ED LOS, which might increase morbidity and mortality [43].

Our results indicate that hospital length of stay is increased, and similar results have also been reported in the acute medical unit setting [40]. More evidence is required to assess resource utilisation, re-visitation rates and ICU admissions for patients with NSC. Further research on cost-effectiveness regarding resource utilisation in the emergency setting versus the admission ward is required. Re-visitation rates merit further study, not only in the light of wasted resources, but also increased mortality: one study found that a revisit after 2–3 days for general disability patients was associated with increased mortality [44].

A summary of discharge diagnoses by ICD-10 main category is shown in Table 4. Previous studies have reported discharge diagnosis for selected patients in higher triage categories which are similar to our findings [5, 15, 45].

### Strengths and limitations

The strength of our review lies in the methodology. We have applied documented and well-known protocols for systematic reviews by registering our review to PROSPERO, by following the PRISMA checklist and assessing the quality of included studies by the SIGN checklist and the overall certainty of evidence with GRADE. We have conducted a systematic database search with the aid of a librarian and each study was individually assessed by two researches to reduce the risk of bias.

The main difficulty in our review was that NSC is not a well-defined term in the literature. We attempted to overcome this by utilising several search terms and strategies. During the screening process, we identified two further search terms and conducted an additional search.

NSCs have not so far been widely researched, which is reflected in the relatively small number of included studies. The included studies were heterogeneous in the selection process for the study population and in the reported outcomes. Hence there is some risk of selection and publication bias in this review. The funnel plots we conducted for our outcomes (Additional file 1) appear asymmetric, however we expect this to be due to small number of included studies rather than significant publication bias.

There was some inconsistency and heterogeneity in the included studies which resulted in lower certainty of evidence by GRADE. All included studies were assessed as having low or unclear risk of bias. We concluded that risk of bias across studies is low and there are no apparent limitations that would lower the confidence of the results. We assessed all studies studies using the SIGN checklist for cohort studies. The checklist was not optimal; however, we are not aware of any tools that might have been more suitable for a prognostic review [27].

We regret having to exclude several studies from the BANC group, which are among the most cited in this field. The grounds for this decision lie in their decision to select patients from only two out of five triage categories, which would have been a major confounding factor for our analysis.

Regarding the included studies, Bhalla et al. did not report individual numerical data on their study subjects. The data we have presented is estimated based on the proportional data they provided. We chose to include this study despite the lack of detail because of the large number of participants in their study, making it the largest study in this field that we are aware of [1].

## Conclusions

Patients presenting with a nonspecific complaint have increased mortality, and their care in the emergency department seems to require more time and resources than patients presenting with specific complaints. NSC patients are admitted to the hospital more often and their stay in the hospital is longer. We suggest that NSC should be considered as a core major emergency presentation and that these patients require an established pathway of care.

## Supplementary information

**Additional file 1: Appendix 1.** Database search strategy. **Appendix 2.** SIGN checklist for cohort studies. **Appendix 3.** potentially relevant, excluded studies. **Appendix 4.** Prisma checklist. **Appendix 5.** Triage allocation across studies. **Appendix 6.** Risk of bias across studies. **Appendix 7.** GRADE assessment of certainty of evidence

## Data Availability

The dataset supporting the conclusions of this article is included within the article and its additional files. Included individual articles are available in respective journals’ archives, which might require subscription according to publishers’ policies.

## Abbreviations

BANC: Basel Nonspecific Complaint Study
DGC: Decreased General Condition
ED: Emergency Department
EMS: Emergency Medical Services
ESI: Emergency Severity Index
GRADE: Grading of Recommendations Assessment, Development and Evaluation
ICD-10: International Classification of Diseases, 10th edition
ICU: Intensive Care Unit
LOS: Length of Stay
NHMCS: The National Hospital Ambulatory Medical Care Survey
NSC: Nonspecific complaint
PRISMA: Preferred Reporting Items for Systematic Reviews and Meta-Analyses
PROSPERO: International prospective register of systematic reviews
RCT: Randomised controlled trial
SC: Specific complaint
SIGN: Scottish Intercollegiate Guidelines Network
TRIPOD: Transparent reporting of a multivariable prediction model for individual prognosis or diagnosis
