# Supplementary material for: Nonspecific complaints in the emergency department – a systematic review
Source: Scand J Trauma Resusc Emerg Med. 2020 Jan 28;28:6. doi: 10.1186/s13049-020-0699-y (PMC6986144; doi:10.1186/s13049-020-0699-y)
Supplement: Supplementary file 1 — Additional file 1: Appendix 1. Database search strategy. Appendix 2. SIGN checklist for cohort studies. Appendix 3. potentially relevant, excluded studies. Appendix 4. Prisma checklist. Appendix 5. Triage allocation across studies. Appendix 6. Risk of bias across studies. Appendix 7. GRADE assessment of certainty of evidence [file 13049_2020_699_MOESM1_ESM.docx]

# Appendix 1: Database search strategy

## **Ovid database**

MEDLINE(R) and Epub Ahead of Print, In-Process & Other Non-Indexed Citations and Daily <1946 to January 25, 2019>

| 1. nonspecific complaint emergency department elderly |
| --- |
| 2. limit 1 to five stars |
| 3. nonspecific complaint*.mp. |
| 4. non-specific complaint*.mp. |
| 5. weakness.mp. |
| 6. lethargy.mp. or exp LETHARGY/ |
| 7. mobility limitation.mp. or exp Mobility Limitation/ |
| 8. 3 or 4 or 5 or 6 or 7 |
| 9. exp Emergency Medical Services/ or exp Emergency Service, Hospital/ or emergency service*.mp. |
| 10. 8 and 9 |
| 11. from 15 keep 1 |
| 12. "failure to thrive".mp. or exp Failure to Thrive/ |
| 13. 9 and 12 |
| 14. exp Anorexia/ |
| 15. acopia.mp. |
| 16. decreased mobility |
| 17. 14 or 15 or 16 |
| 18. 17 and 9 |

## **SCOPUS database**

### 25.1.2019

( ( TITLE-ABS-KEY ( "nonspecific complaint*" ) ) OR ( TITLE-ABS-KEY ( "non-specific complaint*" ) ) OR ( TITLE-ABS-KEY ( "mobility limitation*" ) ) OR ( ( TITLE-ABS-KEY ( lethargy* ) OR TITLE-ABS-KEY ( weakness* ) ) ) ) AND ( TITLE-ABS-KEY ( "emergency department*" ) ) AND NOT DBCOLL ( medl )

4.2.2019

## ( ( TITLE-ABS-KEY ( "nonspecific complaint*" ) )  OR  ( TITLE-ABS-KEY ( "non-specific complaint*" ) )  OR  ( TITLE-ABS-KEY ( "mobility limitation*" ) )  OR  ( ( TITLE-ABS-KEY ( lethargy* )  OR  TITLE-ABS-KEY ( weakness* ) ) ) )  AND  ( TITLE-ABS-KEY ( "emergency medical service*" ) )  AND NOT  DBCOLL ( medl )

### 8.7.2019

( TITLE-ABS-KEY ( "emergency department" ) )  AND  ( ( TITLE-ABS-KEY ( "decreased mobility" ) )  OR  ( TITLE-ABS-KEY ( anorexia ) )  OR  ( TITLE-ABS-KEY ( acopia ) ) )

## **Web of Science database**

| **Set** | **Results** | **Save History / Create AlertOpen Saved History** |
| --- | --- | --- |
| # 13 | [96](http://apps.webofknowledge.com/summary.do?product=WOS&doc=1&qid=14&SID=F6MdDYCvxi4UvMFj3sH&search_mode=CombineSearches&update_back2search_link_param=yes) | #12 AND #6  *Indexes=SCI-EXPANDED, SSCI, A&HCI, CPCI-S, CPCI-SSH, BKCI-S, BKCI-SSH, ESCI, CCR-EXPANDED, IC Timespan=1998-2019* |
| # 12 | [27,986](http://apps.webofknowledge.com/summary.do?product=WOS&doc=1&qid=13&SID=F6MdDYCvxi4UvMFj3sH&search_mode=CombineSearches&update_back2search_link_param=yes) | #11 OR #10 OR #9  *Indexes=SCI-EXPANDED, SSCI, A&HCI, CPCI-S, CPCI-SSH, BKCI-S, BKCI-SSH, ESCI, CCR-EXPANDED, IC Timespan=1998-2019* |
| # 11 | [17](http://apps.webofknowledge.com/summary.do?product=WOS&doc=1&qid=11&SID=F6MdDYCvxi4UvMFj3sH&search_mode=GeneralSearch&update_back2search_link_param=yes) | **TOPIC:** (acopia)  *Indexes=SCI-EXPANDED, SSCI, A&HCI, CPCI-S, CPCI-SSH, BKCI-S, BKCI-SSH, ESCI, CCR-EXPANDED, IC Timespan=1998-2019* |
| # 10 | [27,419](http://apps.webofknowledge.com/summary.do?product=WOS&doc=1&qid=10&SID=F6MdDYCvxi4UvMFj3sH&search_mode=GeneralSearch&update_back2search_link_param=yes) | **TOPIC:** (anorexia)  *Indexes=SCI-EXPANDED, SSCI, A&HCI, CPCI-S, CPCI-SSH, BKCI-S, BKCI-SSH, ESCI, CCR-EXPANDED, IC Timespan=1998-2019* |
| # 9 | [552](http://apps.webofknowledge.com/summary.do?product=WOS&doc=1&qid=9&SID=F6MdDYCvxi4UvMFj3sH&search_mode=GeneralSearch&update_back2search_link_param=yes) | **TOPIC:** ("decreased mobility")  *Indexes=SCI-EXPANDED, SSCI, A&HCI, CPCI-S, CPCI-SSH, BKCI-S, BKCI-SSH, ESCI, CCR-EXPANDED, IC Timespan=1998-2019* |
| # 8 | [997](http://apps.webofknowledge.com/summary.do?product=WOS&doc=1&qid=8&SID=F6MdDYCvxi4UvMFj3sH&search_mode=CombineSearches&update_back2search_link_param=yes) | #7 AND #6  *Indexes=SCI-EXPANDED, SSCI, A&HCI, CPCI-S, CPCI-SSH, BKCI-S, BKCI-SSH, ESCI, CCR-EXPANDED, IC Timespan=1998-2019* |
| # 7 | [110,864](http://apps.webofknowledge.com/summary.do?product=WOS&doc=1&qid=7&SID=F6MdDYCvxi4UvMFj3sH&search_mode=CombineSearches&update_back2search_link_param=yes) | #5 OR #4 OR #3 OR #2  *Indexes=SCI-EXPANDED, SSCI, A&HCI, CPCI-S, CPCI-SSH, BKCI-S, BKCI-SSH, ESCI, CCR-EXPANDED, IC Timespan=1998-2019* |
| # 6 | [87,773](http://apps.webofknowledge.com/summary.do?product=WOS&doc=1&qid=6&SID=F6MdDYCvxi4UvMFj3sH&search_mode=GeneralSearch&update_back2search_link_param=yes) | **TOPIC:** (emergency department*)  *Indexes=SCI-EXPANDED, SSCI, A&HCI, CPCI-S, CPCI-SSH, BKCI-S, BKCI-SSH, ESCI, CCR-EXPANDED, IC Timespan=1998-2019* |
| # 5 | [105,419](http://apps.webofknowledge.com/summary.do?product=WOS&doc=1&qid=5&SID=F6MdDYCvxi4UvMFj3sH&search_mode=GeneralSearch&update_back2search_link_param=yes) | **TOPIC:** (mobility limitation*) *OR* **TOPIC:** (weakness*)  *Indexes=SCI-EXPANDED, SSCI, A&HCI, CPCI-S, CPCI-SSH, BKCI-S, BKCI-SSH, ESCI, CCR-EXPANDED, IC Timespan=1998-2019* |
| # 4 | [4,559](http://apps.webofknowledge.com/summary.do?product=WOS&doc=1&qid=4&SID=F6MdDYCvxi4UvMFj3sH&search_mode=GeneralSearch&update_back2search_link_param=yes) | **TOPIC:** (lethargy*)  *Indexes=SCI-EXPANDED, SSCI, A&HCI, CPCI-S, CPCI-SSH, BKCI-S, BKCI-SSH, ESCI, CCR-EXPANDED, IC Timespan=1998-2019* |
| # 3 | [524](http://apps.webofknowledge.com/summary.do?product=WOS&doc=1&qid=3&SID=F6MdDYCvxi4UvMFj3sH&search_mode=GeneralSearch&update_back2search_link_param=yes) | **TOPIC:** (non-specific complaint*)  *Indexes=SCI-EXPANDED, SSCI, A&HCI, CPCI-S, CPCI-SSH, BKCI-S, BKCI-SSH, ESCI, CCR-EXPANDED, IC Timespan=1998-2019* |
| # 2 | [778](http://apps.webofknowledge.com/summary.do?product=WOS&doc=1&qid=2&SID=F6MdDYCvxi4UvMFj3sH&search_mode=GeneralSearch&update_back2search_link_param=yes) | **TOPIC:** (nonspecific complaint*)  *Indexes=SCI-EXPANDED, SSCI, A&HCI, CPCI-S, CPCI-SSH, BKCI-S, BKCI-SSH, ESCI, CCR-EXPANDED, IC Timespan=1998-2019* |
| # 1 | [778](http://apps.webofknowledge.com/summary.do?product=WOS&doc=1&qid=1&SID=F6MdDYCvxi4UvMFj3sH&search_mode=GeneralSearch&update_back2search_link_param=yes) | **TOPIC:** (nonspecific complaint*)  *Indexes=SCI-EXPANDED, SSCI, A&HCI, CPCI-S, CPCI-SSH, BKCI-S, BKCI-SSH, ESCI, CCR-EXPANDED, IC Timespan=1998-2019* |

# Appendix 2: SIGN checklist for cohort studies

Rows 1.4, 1.5, 1.8, 1.9, 1.11, 1.12, and 2.3 were not relevant for assessing a prognostic review and thus were excluded from table.

|  | **Bhalla et al 2014** | **Borodin et al 2017** | **Djärv et al 2015** | **Karakoumis et al 2015** | **LaMantia et al 2010** | **Nemec et al2010** | **Nickel et al 2009** | **Quinn et al2015** | **Rutschmann et al 2005** | **Safwenberg et al 2007** | **Safwenberg et al 2008** | **Sauter et al 2018** | **Vilpert et al 2018** | **Wachelder et al 2017** |
| --- | --- | --- | --- | --- | --- | --- | --- | --- | --- | --- | --- | --- | --- | --- |
| 1.1 The study addresses an appropriate and clearly focused question. | Y | n | y | y | y | n | y | y | n | y | y | y | y | y |
| 1.2 The two groups being studied are selected from source populations that are comparable in all respects other than the factor under investigation. | y | n/a | y | n/a | can't say | n/a | n/a | n/a | n/a | cant say | cant say | y | n/a | y |
| 1.3 The study indicates how many of the people asked to take part did so, in each of the groups being studied. | n/a | n/a | n/a | n/a | n/a | n/a | n/a | n/a | y | n/a | n/a | n/a | n/a | n/a |
| 1.6 Comparison is made between full participants and those lost to follow up, by exposure status. | n/a | n/a | n/a | y | n/a | Can't say | n/a | n/a | n/a | n/a | n/a | n/a | n/a | cant' say |
| 1.7 The outcomes are clearly defined. | y | n | y | y | y | y | y | y | y | y | y | y | y | y |
| 1.10 The method of assessment of exposure is reliable. | y | n | y | y | y | y | y | y | y | y | y | y | y | y |
| 1.13 The main potential confounders are identified and taken into account in the design and analysis. | y | n | y | y | y | can't say | n | y | y | y | n | y | y | y |
| 1.14 Have confidence intervals been provided? | y | n | y | n | y | n | n | y | n | y | y | y | y | y |
| 2.1 How well was the study done to minimise the risk of bias or confounding. High quality (++) / Acceptable (+) / Unacceptable – reject 0 | + | 0 | + | 0 | + | 0 | + | + | 0 | + | + | + | + | + |
| 2.2 Taking into account clinical considerations, your evaluation of the methodology used, and the statistical power of the study, do you think there is clear evidence of an association between exposure and outcome? | y |  | y | can't say | y | can't say | y | y | cant say | y | y | y | y | y |
| 2.4 Notes. Summarise the authors conclusions. Add any comments on your own assessment of the study, and the extent to which it answers your question and mention any areas of uncertainty raised above. | Large data set, but it is based on visits not individual patients | Poster abstract, very limited information regarding methods | Simultaneously introduced a new triage system | The study excluded patients in the ESI 4-5 category and also excluded patients with serious vital sign deviations. | Limitations, study methods and results were cleary stated. Retrospective study. Population characteristics described adequately | The objective of the study was to describe the patient group with NSC. Also the study left out ESI 4-5 patients. | Peer reviewed clinical letter, concise and clear. Missing discussion of limitations | inter-rater reliability taken into account | Study objective not clearly defined. Reported results lack statistical parameters | Study question, methdods cleary described. Population characteristics not described | The selection of the reference population was explained well. Results were clearly stated. There were also to two researchers analyzing the complaint. Limitations are missing. | Clear objective, sound methodology and reporting. Study protocol published separately | nursing home residents were excluded from the study | Clear objective, sound methodology and reporting |

# Appendix 3: potentially relevant, excluded studies

| Bingisser et al 2017, Weigel et al 2017: These study assess the predictive value of presenting symptoms to the ED. In their analysis, they have considered several symptoms per patient, resulting in a major confounding factor to our analysis that is designed for single main complaint of NSC. |
| --- |
| Borodin et al 2017: This conference abstract met our inclusion criteria, but due to the nature of the short publication format, did not report enough details to meet our quality standard. We look forward to the possibility of reading the full study in the future. |
| Castren et al 2015: This study focuses on Nonspecific complaints in the prehospital environment, and their structural factors. Their study did not include outcomes that were specified in our inclusion criteria |
| Christensen et al 2016 and Sovso et al 2018: While these studies report mortality rates and discharge diagnosi for NSC patients, they have included paediatric data. No stratification by age was available for the outcomes and thus we were unable to include their results. |
| Karakoumis et al 2015: This study has only included patients from two out of five triage categories, which would provide a major confounding factor to our epidemiological analysis. The study lacked a comparison group for other outcomes. |
| Malinovska et al 2018: This study has divided patients into subgroups. We were unable to find other studies with similar subgrouping for comparison. |
| Nemec et al 2010: This study has described the framework for NSC studies. However it only presents patients from selected triage groups, and lacks statistical analysis. |
| Nickel et al 2009: This peer-reviewed letter deserves credit for clarifying the difference between generalised and localised weakness. However, the sample is relatively small and the study population has been selected, which confounds the epidemiological data. |
| Nielsen et al 2018. Nielsen et al have published an enlightening study of general disability patients, however the study population was AMU patients, which means it did not meet our inclusion criteria. |
| Ruger et al 2007: We were interested to read this study of middle acquity patients presenting with weakness. The study includes paediatric data, due to which we have had to exclude it from our analysis. |
| Rutschmann et al 2005: As a descriptive study, this study does not present statistical parameters for the outcomes we have selected for our review. |
| Wallgren et al 2016: This retrospective study looks at septic patients and their outcomes, with a focus for NSC. We included this study in our discussion but had to exclude it from our analysis due to the study setting differing markedly from included studies. |

# Appendix 4: Prisma checklist

| **Section/ topic** | **#** | **Checklist item** | **Reported on page #** | |
| --- | --- | --- | --- | --- |
| **TITLE** | | | |  |
| Title | 1 | Identify the report as a systematic review, meta-analysis, or both. | 1 | |
| **ABSTRACT** | | | |  |
| Structured summary | 2 | Provide a structured summary including, as applicable: background; objectives; data sources; study eligibility criteria, participants, and interventions; study appraisal and synthesis methods; results; limitations; conclusions and implications of key findings; systematic review registration number. | 2 | |
| **INTRODUCTION** | | | |  |
| Rationale | 3 | Describe the rationale for the review in the context of what is already known. | 3-5 | |
| Objectives | 4 | Provide an explicit statement of questions being addressed with reference to participants, interventions, comparisons, outcomes, and study design (PICOS). | 5 | |
| **METHODS** | | | |  |
| Protocol and registration | 5 | Indicate if a review protocol exists, if and where it can be accessed (e.g., Web address), and, if available, provide registration information including registration number. | 5 | |
| Eligibility criteria | 6 | Specify study characteristics (e.g., PICOS, length of follow-up) and report characteristics (e.g., years considered, language, publication status) used as criteria for eligibility, giving rationale. | 5 | |
| Information sources | 7 | Describe all information sources (e.g., databases with dates of coverage, contact with study authors to identify additional studies) in the search and date last searched. | 6 | |
| Search | 8 | Present full electronic search strategy for at least one database, including any limits used, such that it could be repeated. | Appx 1 | |
| Study selection | 9 | State the process for selecting studies (i.e., screening, eligibility, included in systematic review, and, if applicable, included in the meta-analysis). | 6-7 | |
| Data collection process | 10 | Describe method of data extraction from reports (e.g., piloted forms, independently, in duplicate) and any processes for obtaining and confirming data from investigators. | 6-7 | |
| Data items | 11 | List and define all variables for which data were sought (e.g., PICOS, funding sources) and any assumptions and simplifications made. | 6-7 | |
| Risk of bias in individual studies | 12 | Describe methods used for assessing risk of bias of individual studies (including specification of whether this was done at the study or outcome level), and how this information is to be used in any data synthesis. | 8 | |
| Summary measures | 13 | State the principal summary measures (e.g., risk ratio, difference in means). | 8 | |
| Synthesis of results | 14 | Describe the methods of handling data and combining results of studies, if done, including measures of consistency (e.g., I^2^) for each meta-analysis. | 8 | |

| **Section/topic** | **#** | **Checklist item** | **Reported on page #** | |
| --- | --- | --- | --- | --- |
| Risk of bias across studies | 15 | Specify any assessment of risk of bias that may affect the cumulative evidence (e.g., publication bias, selective reporting within studies). | 15, Appx6-7 | |
| Additional analyses | 16 | Describe methods of additional analyses (e.g., sensitivity or subgroup analyses, meta-regression), if done, indicating which were pre-specified. | n/a | |
| **RESULTS** | | | |  |
| Study selection | 17 | Give numbers of studies screened, assessed for eligibility, and included in the review, with reasons for exclusions at each stage, ideally with a flow diagram. | fig1 | |
| Study characteristics | 18 | For each study, present characteristics for which data were extracted (e.g., study size, PICOS, follow-up period) and provide the citations. | Table 2 | |
| Risk of bias within studies | 19 | Present data on risk of bias of each study and, if available, any outcome level assessment (see item 12). | Appx2 | |
| Results of individual studies | 20 | For all outcomes considered (benefits or harms), present, for each study: (a) simple summary data for each intervention group (b) effect estimates and confidence intervals, ideally with a forest plot. | Fig 2-4 | |
| Synthesis of results | 21 | Present results of each meta-analysis done, including confidence intervals and measures of consistency. | Fig 2-4 | |
| Risk of bias across studies | 22 | Present results of any assessment of risk of bias across studies (see item 15) | Appx 7 | |
| Additional analysis | 23 | Give results of additional analyses, if done (e.g., sensitivity or subgroup analyses, meta-regression [see Item 16]). | n/a | |
| **DISCUSSION** | | | |  |
| Summary of evidence | 24 | Summarize the main findings including the strength of evidence for each main outcome; consider their relevance to key groups (e.g., healthcare providers, users, and policy makers). | Appx 7 | |
| Limitations | 25 | Discuss limitations at study and outcome level (e.g., risk of bias), and at review level (e.g., incomplete retrieval of identified research, reporting bias) | 15-16 | |
| Conclusions | 26 | Provide a general interpretation of the results in the context of other evidence, and implications for future research. | 16 | |
| **FUNDING** | | | |  |
| Funding | 27 | Describe sources of funding for the systematic review and other support (e.g., supply of data); role of funders for the systematic review. | 1 | |

# Appendix 5: Triage allocation across studies

|  | **Comment** | **1/red/urgent** | **2/orange/urgent** | **3/yellow/moderate** | **4/green/low** | **5/blue** |
| --- | --- | --- | --- | --- | --- | --- |
| **Sauter et al 2018** | Level of triage p=0,282; consequence of complaint specificity. Triage (univariate regression p=0,101) | NSC 10 (6,1%); SC 32 (5,9%) | NSC 58 (35,2%); SC 241 (44,1%) | NSC 89 (53,9%); SC 255 (46,7%) | NSC 4 (4,2%); SC 14 (2,6%) | NSC 1 (0,6%); SC 4 (0,7%) |
| **Wachelder et al 2017** | Level of triage (%) p=0,30 | NSC 20 (8,2%); SC 188 (12,2%) | | NSC 138 (56,6%); SC 823 (53,4%) | NSC 85 (34,8%); SC 518 (33,6%) |  |
| **Djärv et al 2015** | Triage category p<0,01. Risk of in-hospital death (OR) | NSC 24 (2%); SC 2252 (11%); OR 3,72 (95%CI 1,48-9,37) | NSC 146(12%); SC 6107(29%); OR 10,05 (95%CI 6,60-15,32) | NSC 680 (58%); SC 8807(42%); OR 7,76 (95%CI 5,65-10,67) | NSC 326 (28%); SC 3359 (16%); OR 20,34 (95%CI 10,72-38,59) | NSC 2(0%); SC 250(1%) |
| **Quinn et al 2015** | CTAS 1 or 2 did not predict admission. OR 0,61 (0,08-4,95) | 7(1,46%) | 58(12,1%) | 362(75,7%) | 49(10,2%) | 1(0,2%) |

# Appendix 6: Risk of bias across studies

#
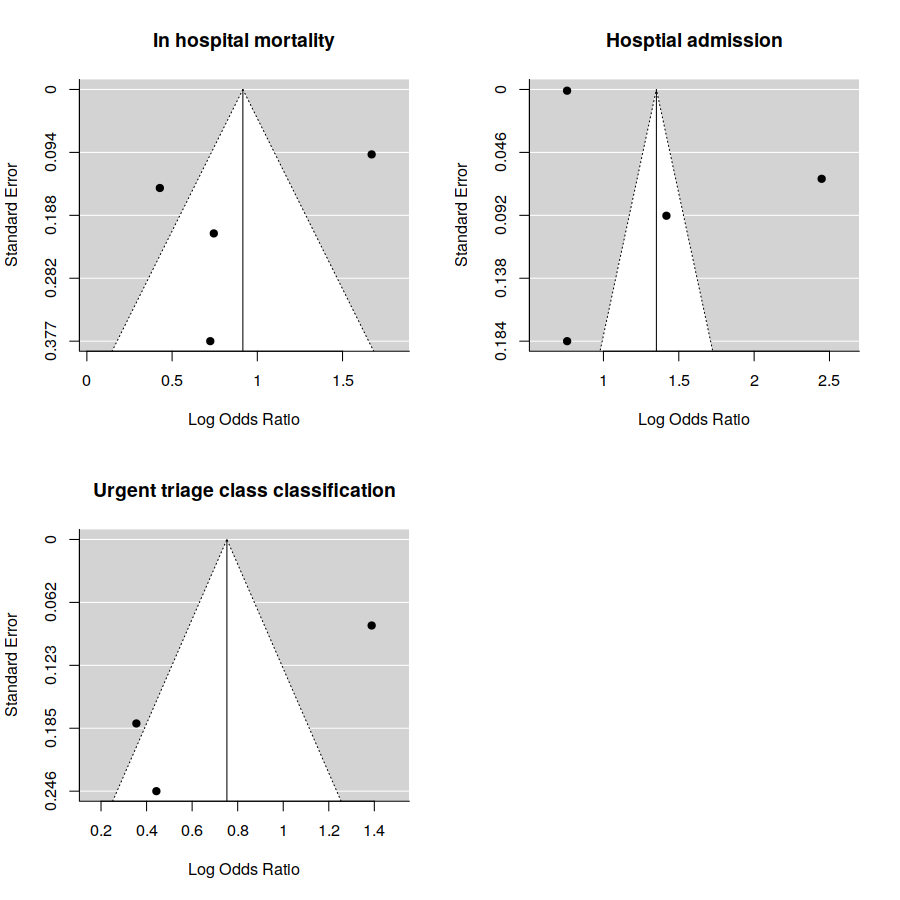


# Appendix 7: GRADE assessment of certainty of evidence
